# Insights into* Brevibacillus borstelensis* AK1 through Whole Genome Sequencing: A Thermophilic Bacterium Isolated from a Hot Spring in Saudi Arabia

**DOI:** 10.1155/2018/5862437

**Published:** 2018-05-24

**Authors:** Amjad B. Khalil, Neelamegam Sivakumar, Muhammad Arslan, Hamna Saleem, Sami Qarawi

**Affiliations:** ^1^Department of Life Sciences, King Fahd University of Petroleum and Minerals, Dhahran, Saudi Arabia; ^2^Biosciences Core Lab, King Abdullah University of Science and Technology, Thuwal, Jeddah, Saudi Arabia; ^3^Environmental Biotechnology Division, National Institute for Biotechnology and Genetic Engineering, Faisalabad, Pakistan; ^4^Department of Environmental Biotechnology, Helmholtz Centre for Environmental Research, Leipzig, Germany; ^5^Institute of Molecular Biology and Biotechnology, The University of Lahore, Lahore, Pakistan

## Abstract

*Brevibacillus borstelensis *AK1 is a thermophile which grows between the temperatures of 45°C and 70°C. The present study is an extended genome report of* B. borstelensis *AK1 along with the morphological characterization. The strain is isolated from a hot spring in Saudi Arabia (southeast of the city Gazan). It is observed that the strain AK1 is rod-shaped, motile, and strictly aerobic bacterium. The whole genome sequence resulted in 29 contigs with a total length of 5,155,092 bp. In total, 3,946 protein-coding genes and 139 RNA genes were identified. Comparison with the previously submitted strains of* B. borstelensis *strains illustrates that strain AK1 has a small genome size but high GC content. The strain possesses putative genes for degradation of a wide range of substrates including polyethylene (plastic) and long-chain hydrocarbons. These genomic features may be useful for future environmental/biotechnological applications.

## 1. Introduction

Thermophiles are a group of heat-loving microorganisms which have an optimum growth temperature of at least 50°C [1]. The genus* Brevibacillus* (family* Paenibacillaceae *and class Bacilli) was initially described as* Bacillus brevis *in 1900 by Migula [2]. In later years, many new strains were placed in the same group (e.g.,* B. brevis*) including the strains that were not following the* sensu stricto *criteria that challenged the overall classification. This included discrepancies in maximum growth temperatures and a wide range of GC values, hence confirming* B. brevis *as a heterogeneous group [3, 4]. To resolve these ambiguities, a new genus named* Brevibacillus *was proposed in 1996 that resulted in reclassification of nearly 10* Bacillus *species, based on 16S rDNA analysis [5, 6]. Since then, many strains have been reclassified as novel species of the genera originally reported as members of* Bacillus brevis *[6–9]. The genus* Brevibacillus *comprised environmental bacteria that have been observed in diverse habitats including agricultural soil, wastewaters, and hot springs [10, 11].

Earlier studies have shown the importance of* B. borstelensis *in different spheres of industry and the environment [23–26]. For instance, Arya et al. [25] reported that* B. borstelensis *possess great potential to degrade the fungicide carbendazim from agricultural fields at high rates, especially when coupled with* Streptomyces albogriseolus*. Hadad et al. [24] showed that inert polyethylene could be degraded by* B. borstelensis *strain 707. Tsai et al. [26] evinced that the lipolytic* B. borstelensis *strain SH168 can enhance the transformation of food wastes into biofertilizer. Baek et al. [23] reported that* B. borstelensis *strain BCS-1 could produce D-amino acid amidases, a catalyst for the formation of optically pure D-amino acids; these are the intermediates of pharmaceuticals production, food additives, insecticides, synthetic sweeteners, and agrochemicals [27]. Furthermore, an alkaline pectin lyase applicable in fruit juice and oil extract has also been isolated from* B. borstelensis *P35. The reassociation analyses, cellular fatty acid profile, and isoprenoid quinone composition analysis confirmed that* B. borstelensis *owns these properties based on unique DNA base compositions [6, 7].

To date, 23 species of the genus* Brevibacillus *have been recognized including* B. borstelensis*. Nevertheless, only four strains of* B. borstelensis *are subjected to whole genome sequencing so far. These are* B. borstelensis *cifa_chp40 [PRJNA200540],* B. borstelensis *3096-7 [NZ_JAQG01000000],* B. borstelensis *LChuR05 [PRJNA271204], and* B. borstelensis *AK1 [PRJNA191598]. In this study, we report the genomic insights of* B. borstelensis *strain AK1 that may help unravel the potential importance of the species in biotechnology. Additionally, since the strain has been previously reported for potential polyethylene (plastic) degradation, we identified the putative genes/enzymes based on KEGG orthology.

## 2. Materials and Methods

### 2.1. Sampling

The sampling was performed in January 2012 at a hot spring “Al-Ain Alhara”, located in the southeast of Gazan city in Saudi Arabia (16°56′N, 43°15′E). Water samples were taken in sterile thermal glass containers while maintaining the physicochemical quality parameters.

### 2.2. Growth Conditions and Genomic DNA Preparation

A 5 ml aliquot of the water sample was inoculated in 250 ml of tetrathionate (TT) broth (ATCC medium 697). Incubation was performed at 55°C for 24 to 48 h at shaking speed of 300 rpm. The bacterial cells were then harvested by centrifugation (14,000 g) and the pellet was spread over TT agar media. Statistically relevant numbers of distinctive colonies were picked and the procedure was repeated thrice to purify the strain for subsequent genomic analysis.

### 2.3. Morphological Characterization

For morphological characterization, bacterial cells were obtained from stationary phase and subjected to SEM microscopy. Briefly, fixation was performed in 2.5% glutaraldehyde, which was buffered at pH 7.2 with phosphate buffer saline (PBS). Subsequently, the mixture was placed over ice for 2 h followed by washing of bacterial cells for 20 min at room temperature. The post-fixation was performed in osmium tetraoxide (1%) leading to dehydration in a graded ethanol series up to 100% concentration. Lastly, the specimens were gold-plated (10 nm) and studied under a field emission SEM.

### 2.4. DNA Extraction

Total bacterial DNA extraction was performed using Genomic DNA isolation kit (Norgen Biotek) as per the manufacturer's guidelines. DNA yield was measured using Qbit Assay that was approximately 40 *μ*g, while the quality was determined using Bioanalyzer and Agarose gel before proceeding to library preparation for sequencing.

### 2.5. Genome Sequencing, Assembly, and Functional Annotation

The genome sequencing was performed using a 454-genome sequencer (FLX titanium, Roche), based at Bioscience Core Laboratory of King Abdullah University of Science and Technology (KAUST) in Saudi Arabia. Briefly, 500 ng of genomic DNA was used to construct the fragment library using Rapid Library Preparation Kit. Average insert sizes of the yielded libraries were 750 bp in length. These libraries were then quantified by qPCR. The sequences were subjected to quality control to trim low-quality reads. The filtered sequences were assembled into contigs using Newbler assembler v2.5.3. The automated assembly algorithm yielded over 100 contigs. Out of these contigs, 29 were selected based on high contig length as they represented the expected whole genome length. The assembly was then annotated using Prokaryotic Genome Automatic Annotation Pipeline (PGAAP) available at NCBI. The annotation yielded a total of 5,090 coding genes and out of these 4,951 appeared to be protein-coding genes. Additionally, gene prediction analyses were completed within Integrated Microbial Genomes Expert Review (IMG-ER). The circular visualization of the assembled contigs was generated using an online web-server Circos plotting tool (ClicOFS) [22]. The project summary, as well as associated MIGS information, is shown in Table 1.

### 2.6. Phylogenetic Analysis

For phylogenetic analysis, the sequence of the 16S rRNA gene was aligned with equivalent 16S rRNA genes of closely related strains as appeared in BLAST search. The tree was calculated with an improved neighbor-joining algorithm known as BioNJ.

### 2.7. Accession Numbers

The sequenced genome is deposited in GenBank with master record accession no. APBN00000000: the associated 29 contigs are assigned IDs according to this record (accession numbers APBN01000001 to APBN01000029).

## 3. Results and Discussion

### 3.1. Morphological Features and Classification

**Figure 1:**
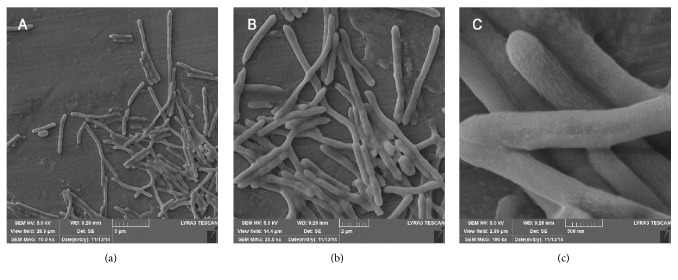
Scanning electron micrographs of* Brevibacillus borstelensis* strain AK1 at 10.0 kx (a), 20.0 kx (b), and 100.0 kx (c).

**Figure 2:**
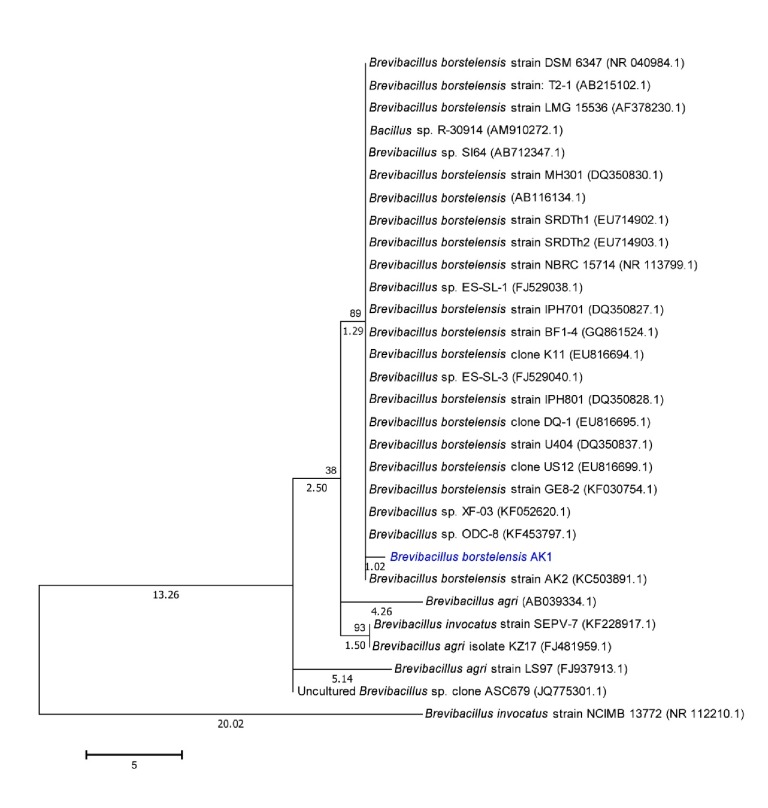
Molecular Phylogenetic analysis by Maximum Likelihood method highlighting the position of* Brevibacillus borstelensis* AK1 relative to other similar bacteria. Bootstrap values based on 1000 replicates show the robustness of the branching. Scale bar represents 0.1 substitutions per nucleotide position. The tree is drawn to scale, with branch lengths measured in the number of substitutions per site.

Morphological analysis revealed that, in the stationary phase, average diameter of* B. borstelensis *AK1 ranged from 0.2 to 0.5 *μ*m whereas the length varied between 2.0 *μ*m and 15.0 *μ*m (Figure 1). The cells appeared to be fast growing, forming colonies of 3 mm diameter within a period of 24 hours. The average generation time was recorded to be 30 min at previously described conditions. The colonies were yellow-pigmented and had smooth margins while the strain was able to withstand a pH range of 5.5–8.5. The general feature information as suggested by Minimum Information about the Genome Sequence (MIGS) is presented in Table 2. Likewise, phylogenetic analysis displayed that the strain was closely related to* B. borstelensis *AK2 and* Brevibacillus *sp. ODC-8 (Figure 2).

### 3.2. Genome Properties

**Figure 3:**
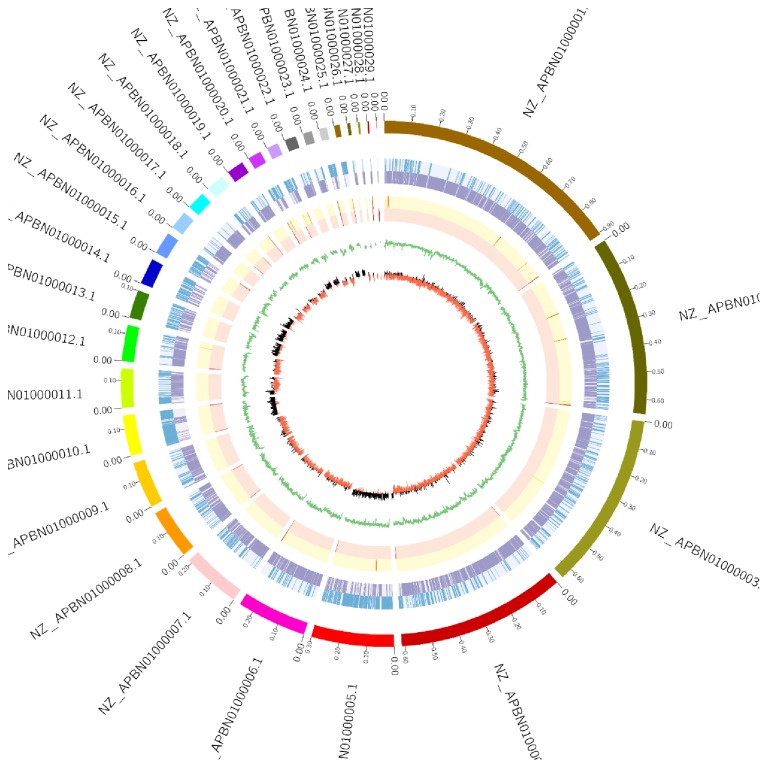
Graphical circular map drawn using ClicO FS [22]. The outer circle represents contigs information, whereas the next two inner circles present ORFs oriented in the forward (blue) and reverse (purple) direction. The 4th circle marks tRNA gene operon (orange) while 5th circle represents rRNA genes (red). The sixth circle reflects GC content plot (%age). The innermost circle shows GC skew; red indicates negative values, whereas black indicates positive values. The scale bar is in Mb.

A genome sequence of 5,155,092 bp with 52% of GC content was produced out of 29 contigs of* B. borstelensis *AK1 (Figure 3; Table 3). More precisely, this comprised 5,090 predicted genes, of which 4,951 (97.27%) were protein-coding genes while 139 were RNA genes (2.73%). Among these RNA genes, 22 belonged to rRNA that contained eleven 16S, ten 23S, and one 5S gene; on the other hand, 117 genes appeared to be tRNA genes. The putative function was assigned to 3,921 genes (77.03%) whereas the remaining genes were annotated as hypothetical proteins. Protein-coding genes connected to KEGG pathways were 1,348 (26.48%) while genes associated with COG categories appeared to be 3,231 (63.48%). The genes distribution into COGs functional categories is displayed in Table 4.

### 3.3. Genomic Insights and Comparative Analysis

To date, four strains of* B. borstelensis *have been sequenced whose genome information is available in the IMG-JGI database. This includes* B. borstelensis *strain 3096-7,* B. borstelensis *strain cifa_chp40,* B. borstelensis *strain LChuR05, and* B. borstelensis *strain AK1. The strain LChuR05, however, has been found to be contaminated and the record has been removed from the GenBank. Hereby, we compare the genome sequence of* B. borstelensis *strain AK1 with the other three strains as described above. The physical comparison shows that the genome sequence of strain AK1 (5.155 Mb) is nearly equal to the strain cifa_chp40, i.e., 5.19 Mb; nevertheless, it is the smallest as compared to the other strains. By contrast, the strain 3096-7 displayed the biggest genome size, i.e., 5.46 Mb. The GC content of the strain AK1 genome is the highest (52.0%) when compared to the other strains of* B. borstelensis*, i.e., strain 3096-7 of 51.4% and strain cifa_chp40 of 51.9%. The gene content of the strain AK1 is the smallest; and likewise, it has the lowest number of protein-coding genes (4,951) followed by the strains cifa_chp40 and 3096-7 (5,042 and 5,352, respectively). Among these genes, AK1 had 3,921 protein-coding genes with function prediction, which is similar to the strain cifa_chp40 that had 3,922 genes. The strain 3096-7, however, had 4,073 genes which is still consistent with these numbers. Further investigations on protein-coding genes and enzymes illustrated the presence of 1,208 genes for the strain AK1, 1,221 genes for strain 3096-7, and 1,215 genes for the strain cifa_chp40. The detailed description of these parameters along with additional features is presented in Table 5. The pairwise average nucleotide identity (ANI) values of all the sequenced strains of* B. borstelensis *are presented in Table 6. It is found that all of the strains have similar ANI values ranging from 99.51% to 99.58%.

### 3.4. Putative Genes Involved in Degradation Services: Metagenomic Assessment

We further attempted to identify putative genes/enzymes of the strain AK1 based on KEGG orthology, KO (i.e., enzyme commission classification), that may possess a potential in degradation services including polyethylene and hydrocarbons. We found presence of 1 cutinase, 67 lipases, 99 hydroxylases, 2 laccases, 1 polyphenol oxidase, and 116 proteases KO database (Supplementary Data) (available here). All of them have been previously reported as potential plastic degrading genes in different bacteria [28–34]. Similarly, 159 monooxygenases, 136 dioxygenases, and 118 lyases are identified (Supplementary Data). These enzymes appeared to have a potential role in a number of bioremediation studies including catabolic expression of CYP family [35–37]. In any case, the results strengthen the significance of strain AK1 for future biotechnological services that come with advantage of extremophile properties (i.e., high GC content).

## 4. Conclusions

Thermophilic organisms are not only of industrial importance, but they can also be exploited in pollutant degradation services.* B. borstelensis *AK1 was selected based on such a potential and the whole genome sequencing was performed to unravel genomic insights. A general comparison with previously sequenced strains of the same species revealed that the strain AK1 has the smallest genome size but highest GC content. Nevertheless, the presence of putative biodegradation related genes supports the idea of exploiting the species in future environmental/biotechnological services.

## Figures and Tables

**Table 1:** The summary of the project information.

**MIGS ID**	**Property**	**Term**
MIGS-31	Finishing quality	Complete - High-quality draft
MIGS-28	Libraries used	454 shotgun libraries
MIGS-29	Sequencing platforms	454-GS-FLX
MIGS-31.2	Fold coverage	26.52x
MIGS-30	Assemblers	Newbler v. 2.5.3
MIGS-32	Gene calling method	Glimmer
	Genbank Accession	APBN00000000
	Genbank Date of Release	2013-11-05
	GOLD ID	Gi0043156
MIGS-13	Project relevance	Industrial

**Table 2:** Classification and general features of *B. borstelensis* strain AK1 according to the MIGS recommendations.

MIGS ID	Property	Term	Evidence Code
	Current Classification	Domain *Bacteria*	TAS [12]
		Phylum *Firmicutes*	TAS [13–15]
		Class *Bacilli*	TAS [16, 17]
		Order *Bacillales*	TAS [18]
		Family *Paenibacillaceae*	TAS [19]
		Genus *Brevibacillus *	TAS [20, 21]
		Species *borstelensis*	TAS [21]
		Strain: AK1	TAS [In this report]
	Gram stain	Positive	TAS [In this report]
	Cell shape	Rod	TAS [In this report]
	Motility	Motile	TAS [In this report]
	Sporulation	Spore forming	TAS [In this report]
	Temperature range	40–70°C	TAS [In this report]
	Optimum pH	7.5	TAS [In this report]
	Optimum temperature	45–50°C	TAS [In this report]

	pH range	6–8	TAS [In this report]

MIGS-22	Carbon source	Maltose, cellobiose, d-fructose, d-galactose, d-glucose lactose, lactulose, d-mannose, sucrose, trehalose, d-xylose	TAS [In this report]

MIGS-6	Habitat	Hot spring	TAS [In this report]

MIGS-6.3	Salinity	No growth with > 1% NaCl (w/v)	TAS [In this report]

MIGS-15	Biotic relationship	Free-living	TAS [In this report]

MIGS-14	Pathogenicity	Non-pathogen	TAS [In this report]

MIGS-4	Geographic location	50 km southeast of Gazan, Saudi Arabia	TAS [In this report]

MIGS-5	Collection date	January 2012	NAS [In this report]

MIGS-4.1	Latitude	16°56′N	TAS [In this report]

MIGS-4.2	Longitude	43°15′E	TAS [In this report]

Evidence codes - IDA: Inferred from Direct Assay; TAS: Traceable Author Statement (i.e., a direct report exists in the literature); NAS: Non-traceable Author Statement (i.e., not directly observed for the living, isolated sample, but based on a generally accepted property for the species, or anecdotal evidence). These evidence codes are from the Gene Ontology project. If the evidence is IDA, then the property was directly observed for a live isolate by one of the authors or an expert mentioned in the acknowledgements.

**Table 3:** Nucleotide and gene content of the genome.

Attribute	Genome (total)	
	Value	% of total^a^
Size (bp)	5155092	100.00
G+C content (bp)	2680425	52.00
Coding region (bp)	4471011	86.73
Total genes^b^	5090	100.00
RNA genes	139	2.73
Protein-coding genes	4951	97.27
Genes with function predictions	3946	77.52
Protein coding genes with enzymes	1186	23.30
Genes assigned to COGs	3231	63.48
COG clusters	1677	51.90
Genes with signal peptides	303	5.95
Genes with transmembrane helices	1366	26.84
Fused protein coding genes	88	1.73

The total is based on either the size of the genome in base pairs or the total number of protein coding genes in the annotated genome.

**Table 4:** Number of genes associated with the 25 general COG functional categories.

Code	Value	% of total	Description
J	245	3.97	Translation
A	25	0.41	RNA processing and modification
K	231	3.75	Transcription
L	238	3.86	Replication, recombination and repair
B	19	0.31	Chromatin structure and dynamics
D	72	1.17	Cell cycle control, mitosis and meiosis
Y	2	0.03	Nuclear structure
V	46	0.75	Defense mechanisms
T	152	2.46	Signal transduction mechanisms
M	188	3.05	Cell wall/membrane biogenesis
N	96	1.56	Cell motility
Z	12	0.19	Cytoskeleton
W	1	0.02	Extracellular structures
U	158	2.56	Intracellular trafficking and secretion
O	203	3.29	Posttranslational modification, protein turnover, chaperones
C	258	4.18	Energy production and conversion
G	230	3.73	Carbohydrate transport and metabolism
E	270	4.38	Amino acid transport and metabolism
F	95	1.54	Nucleotide transport and metabolism
H	179	2.9	Coenzyme transport and metabolism
I	94	1.52	Lipid transport and metabolism
P	212	3.44	Inorganic ion transport and metabolism
Q	88	1.43	Secondary metabolites biosynthesis, transport and catabolism
R	702	11.38	General function prediction only
S	1347	21.84	Function unknown
-	1005	16.29	Not in COGs

**Table 5:** Genome report for four strains of *Brevibacillus borstelensis* submitted to GenBank.

Organism Name	Size (Mb)	GC%	Scaffolds	Total Genes	Proteins	Protein coding genes	RNA genes	Protein coding genes with function prediction	Protein coding genes with enzymes	Protein coding genes with COGs3	Chromosomal cassette	COG Clusters	Symmetric Identity of AK1 with other strains (%)
*Brevibacillus borstelensis* AK1	5.1550	52	29	5037	4817	4951	139	3946	1208	3231	493	1677	-

*Brevibacillus borstelensis* 3096-7	5.4642	51.4	192	5302	5112	5352	129	4073	1221	3322	593	1709	91.56

*Brevibacillus borstelensis* cifa_chp40	5.1965	51.9	38	5086	4918	5042	135	3922	1215	3270	487	1693	94.72

**Table 6:** Genomic comparisons of different strains of *B. borstelensis *using ANI (in %age).

	*B. borstelensis *AK1	*B. borstelensis *3096-7	*B. borstelensis* cifa_chp40
*B. borstelensis* AK1	100.00	99.51	99.58
*B. borstelensis *3096-7	99.51	100.00	99.52
*B. borstelensis *cifa_chp40	99.58	99.52	100.00

## Supplementary Materials

Supplementary Materials A list of putative genes (entries) of the strain AK1 based on KEGG orthology, KO (i.e., enzyme commission classification), that may possess a potential in degradation of polyethylene, hydrocarbons, and so forth.
